# An incidental intracranial lesion in a young woman with head trauma: The key role of CT bone reconstruction in the diagnosis of an osteoma of the anterior cranial fossa

**DOI:** 10.1002/ccr3.2049

**Published:** 2019-02-11

**Authors:** Grigorios Gkasdaris, Theodossios Birbilis, Orestis Ioannidis, Konstantinos Tsalis

**Affiliations:** ^1^ Fourth Surgical Department, Medical School Aristotle University of Thessaloniki Thessaloniki Greece; ^2^ Department of Neurosurgery University Hospital of Alexandroupolis, Democritus University of Thrace, Medical School Alexandroupolis Greece

**Keywords:** anterior cranial fossa, brain, ct, osteoma, reconstruction, skull base

## Abstract

A young female was subjected to brain CT due to head trauma after a car accident. A lesion was found in the area of the right frontal lobe raising concern. After reconstruction of the CT slices, the radiologic features indicated a skull base osteoma, without the need for further intervention.

## 1 CASE DESCRIPTION

A 27‐year‐old female patient was presented to our emergency room with post‐traumatic amnesia and pain in the abdomen after a reported car accident. Hemodynamically‐respiratorically stable, GCS 15∕15, normal papillary response, tetrakinetic, without any neurologic deficit. CT scan findings suggested mild spleen injury, and fractures of the L1, L2 without dislocation or compression of the spinal canal. She was admitted to our surgical department for monitoring and conservative treatment with lumbar brace. In the brain ct, a round bone density extra‐axial mass (1.1 cm) was depicted in the area of the right frontal lobe.

**Figure 1:**
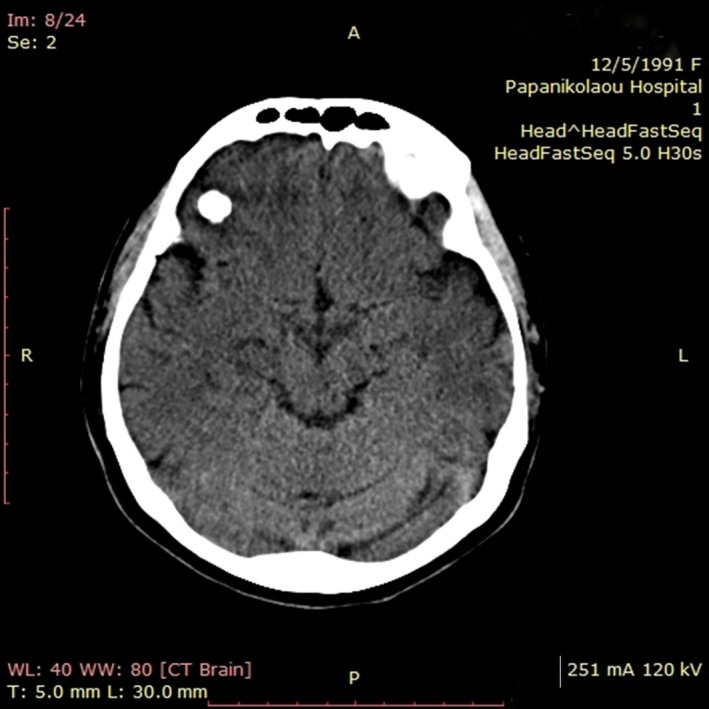
Axial brain CT scan depicting a bone density mass in the right frontal lobe region

**Figure 2:**
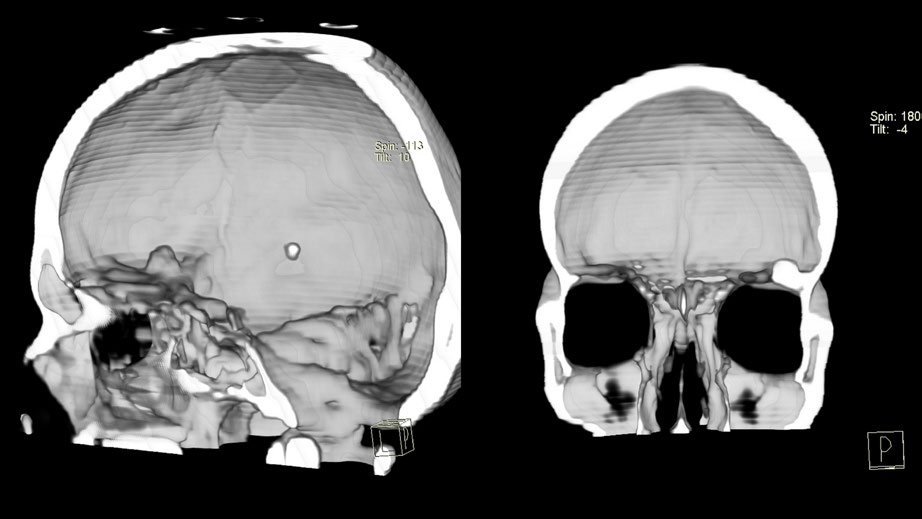
3D CT reconstruction of the skull depicting the mass located in the right anterior cranial fossa

The differential diagnosis was problematic due to the fact that the lesion was small and the slices thick (5 mm) because the CT was performed as routine examination in order to exclude any major traumatic finding. After reconstruction, the radiologic features indicated an intracranial osteoma of the right anterior cranial fossa (Figures 1 and 2).

Osteomas of the skull base are rare. Their clinical presentation can vary depending on location and size. CT is the preferred imaging method. They are usually seen as a homogenous hyperdense mass. Differential diagnosis includes various types of benign bone tumors, epidermoid tumor, calcified meningioma, extra‐axial gliomas, parasite infection, and post‐traumatic porencephaly. Management can be surgically challenging in large osteomas.^1^, ^2^ Due to the small size of the lesion and the absence of symptoms, short‐term follow‐up was decided in our case.

## CONFLICT OF INTEREST

The authors have none to declare.

## AUTHOR CONTRIBUTION

GG: wrote the manuscript and assisted to the evaluation of the patient's every day condition. TB: critically reviewed the neurosurgical aspect of the paper. OI: supervised the patient's care and management. KT: contributed to critical analysis of the paper.
